# Examining the relationships between parent experiences and youth self-reports of slapping/spanking: a population-based cross-sectional study

**DOI:** 10.1186/s12889-019-7729-6

**Published:** 2019-10-22

**Authors:** Tracie O. Afifi, Janique Fortier, Harriet L. MacMillan, Andrea Gonzalez, Melissa Kimber, Katholiki Georgiades, Laura Duncan, Tamara Taillieu, Isabel Garces Davila, Shannon Struck

**Affiliations:** 1 Departments of Community Health Sciences and Psychiatry, University of Manitoba, S113-750 Bannatyne Avenue, Winnipeg, Manitoba R3E 0W5 Canada; 2 Department of Community Health Sciences, University of Manitoba, Winnipeg, Canada; 3 Departments of Psychiatry and Behavioural Neurosciences, and of Pediatrics, McMaster University, Hamilton, Canada; 4 Department of Psychiatry and Behavioural Neurosciences, McMaster University, Hamilton, Canada; 5 Offord Centre for Child Studies, Department of Psychiatry & Behavioural Neurosciences, McMaster University, Hamilton, Canada; 6 Department of Community Health Sciences, University of Manitoba, Winnipeg, Canada

**Keywords:** Spanking, Child maltreatment, Mental health, Prevention, Adolescents

## Abstract

**Background:**

Slapping/spanking is related to a number of poor health outcomes. Understanding what factors are related to the increased or decreased use of spanking/slapping is necessary to inform prevention. This study used a population-based sample to determine the prevalence of slapping/spanking reported by youth; the relationship between sociodemographic factors and slapping/spanking; and the extent to which parental exposures to victimization and maltreatment in childhood and current parental mental health, substance use and family circumstances, are associated with youth reports of slapping/spanking.

**Methods:**

Data were from the 2014 Ontario Child Health Study, a provincially representative sample of households with children and youth aged 4–17 years. Self-reported lifetime slapping/spanking prevalence was determined using a sub-sample of youth aged 14–17 years (*n* = 1883). Parents/primary caregivers (i.e., person most knowledgeable (PMK) of the youth) self-reported their own childhood experiences including bullying victimization, slapping/spanking and child maltreatment, and current mental health, substance use and family circumstances including mental health functioning and emotional well-being, alcohol use, smoking, marital conflict and family functioning. Analyses were conducted in 2018.

**Results:**

Living in urban compared to rural residence and family poverty were associated with decreased odds of slapping/spanking. PMK childhood experiences of physical and verbal bullying victimization, spanking, sexual abuse, emotional abuse, and exposure to physical intimate partner violence were associated with increased odds of youth reported slapping/spanking (adjusted odds ratio [AOR] ranged from 1.33–1.77). PMK experiences of physical abuse and exposure to emotional/verbal intimate partner violence in childhood was associated with decreased odds of youth reported slapping/spanking (AOR = 0.72 and 0.88, respectively). PMK’s higher levels of marital conflict, languishing to moderate mental health functioning and emotional well-being, and moderate or greater alcohol use were associated with increased odds of youth reported slapping/spanking (AOR ranged from 1.36–1.61).

**Conclusions:**

It may be important to consider parent/primary caregiver’s childhood experiences with victimization and maltreatment along with their current parental mental health, substance use and family circumstances when developing and testing strategies to prevent slapping/spanking.

## Background

Spanking is often defined as “the use of physical force with the intention of causing a child to experience pain, but not injury, for the purpose of correcting or controlling the child’s behaviour.” [1] ^(p3)^ It is well-documented that spanking is associated with a number of adverse outcomes for children including externalizing problems such as antisocial behaviour and aggression; internalizing problems including anxiety and depression; and negative relationships with parents [2, 3]. In addition, there is no evidence that spanking is associated with improved child behaviour [3] or that spanking enhances developmental health [4]. Poor outcomes related to physical punishment are also noted across the lifespan into adulthood [5–9]. Based on the current state of knowledge, researchers have recommended that spanking be considered an Adverse Childhood Experience (ACE) and included among efforts to prevent violence [7]. Most recently, the American Academy of Pediatrics has published an updated policy statement that parents and caregivers should not use corporal punishment (including hitting and spanking) on children and adolescents [10].

It is necessary to help parents find effective ways to replace spanking with positive and non-physical parenting strategies to improve and manage their child’s behaviour [11]. An important area of inquiry to expand our knowledge and to inform such prevention efforts is to examine what parental and family factors are related to an increased or decreased likelihood in the use of spanking. To date, some studies have examined the relationship between maternal sociodemographic factors and the use of spanking. Results indicate that parental education, employment, income adequacy, single parent status, ethnicity, and residence in an urban versus rural community are associated with use of spanking [12–17]. However, there have been inconsistencies in these findings, with some studies failing to find an association between sociodemographic factors like parental education, income, and marital status and the likelihood of spanking [12, 18, 19]. Although these studies are informative and important, it is necessary to expand our knowledge beyond parental sociodemographic factors.

An emerging literature considers parental childhood experiences as a way to better understand attitudes towards spanking. For example, previous research has indicated that adult respondents who report being spanked as children were more likely to report favorable attitudes towards spanking [20, 21], but those experiencing emotional abuse [20], severe physical abuse [20], psychological aggression [21], exposure to violence in the home [21], and violence between parents and siblings were less likely to support the use of spanking [21]. However, these studies have only addressed attitudes towards spanking and not if being spanked as a child is linked with an increased likelihood that one’s own child will be spanked. Furthermore, less is currently known about how a parent’s own experiences of child maltreatment such as sexual abuse, physical neglect, and exposure to intimate partner violence (IPV) or how a parent’s childhood experiences of being bullied by peers may be related to the likelihood of their child being spanked.

A parent’s current mental health status and family processes, including the quality of the marital relationship, family functioning, mental health functioning and emotional well-being, and substance use, are also important factors to consider when understanding the likelihood of spanking within a home. Parental stress has been found to be related to spanking [15, 22], as have lower parental emotional well-being [23], and depression [22, 24]. Additionally, previous research has indicated that spanking and harsh physical punishment are related to substance use in adulthood [5–9]. Although some studies have found a relationship between parents’ substance use and spanking, mainly in young children [22, 25], this relationship has not been extensively studied using representative samples. Having data from representative samples is important for generalizability and informing intervention strategies. Therefore, missing from the current literature is the examination of whether parental mental health, substance use and family circumstances may be related to the use of spanking. Such knowledge can be useful in developing targeted, population-based approaches to reduce the use of spanking.

The current study makes the following contributions. First, it uses a representative sample of adolescents aged 14 to 17 years old from Ontario, Canada. Second, parental experiences of childhood bullying by peers, slapping/spanking, and child maltreatment add novel information for understanding the likelihood of slapping/spanking in their children. Third, parent’s current mental health, substance use and family circumstances were also considered (i.e., marital conflict, family functioning, mental health functioning and emotional well-being, smoking, and alcohol use) in relation to the likelihood of slapping/spanking. Finally, the current study uses youth’s own self-reports of being slapped/spanked rather than reports by parents.

Data for the current study were collected in Ontario, Canada— Canada’s most populous province. Earlier Ontario data from 1990 and 1991 indicated that 33.4 and 5.5% of the adult population reported being slapped/spanked in childhood sometimes or often, respectively [9]. Representative data from 2012 showed that 32% of the adult population in Ontario reported experiencing child abuse including physical abuse, sexual abuse, and/or exposure to intimate partner violence [26]. The objectives of the current study were to determine: 1) the lifetime prevalence of slapping/spanking experienced by parents and self-reported experiences of slapping/spanking by youth aged 14 to 17 years old in Ontario, Canada; 2) whether sociodemographic factors are related to youth’s reports of slapping/spanking; and 3) if parental exposures to victimization and maltreatment in childhood and parental current mental health, substance use and family circumstances including mental health functioning and emotional well-being, alcohol use, smoking, marital conflict, perception of family functioning were associated with an increased likelihood of youth self-reports of being slapped/spanked.

## Methods

### Data and sample

This study used data from the 2014 Ontario Child Heath Study (2014 OCHS) collected from October 2014 to October 2015 in Ontario, Canada (analyzed in 2018). The 2014 OCHS used a three-stage sampling design to randomly select a provincially representative sample of households with children aged 4 to 17 years (*N* = 15,796 dwellings, response rate = 50.8%). Data used in the present study were obtained from one randomly selected child living in each household and the person in the household most knowledgeable (PMK) about the selected child. The majority of the PMKs in the current sample were biological, adoptive, or step mothers (88.4%) with other PMKs were biological, adoptive, or step fathers (9.7%), and related or unrelated male or female caregivers (1.4%). Due to the sensitive nature of the questions and excluding siblings from this study, slapping/spanking questions were asked of selected youth aged 14 to 17 years old (*n* = 1883). The 2014 OCHS was a voluntary survey conducted under the Statistics Act, which provides respondents guarantees of their privacy and confidentially. Parents and children provided their consent to participate. The Hamilton Integrated Research Ethics Board at McMaster University approved the study procedures.

### Measurements

#### PMK childhood experiences

PMKs were asked about their childhood experiences before the age of 16 years. Physical bullying included being pushed around, hit or beaten up by other kids or a group of kids three or more times. For verbal bullying victimization, PMKs who reported being hassled or picked on by other kids who said hurtful or mean things to them six or more times, were categorized as having experienced verbal bullying as children. PMKs were asked about their child maltreatment exposure (physical abuse, sexual abuse, exposure to IPV, emotional abuse, and physical neglect). Child maltreatment exposure was assessed using questions and cut-offs derived from multiple sources [27, 28]. Respondents were categorized as having experienced physical abuse if they experienced one or more of the following: 1) being slapped on the face, head or ears, or spanked with something hard three or more times; 2) being pushed, grabbed or shoved, or having something thrown at the respondent to hurt them three or more times; and 3) being kicked, bit, punched, choked, burned, or physically attacked one or more times. PMKs were categorized as having experienced sexual abuse if they experienced one or more of the following: 1) attempted or being forced into unwanted sexual activity by being threatened, held down or hurt in some way one or more times; and 2) being sexually touched, meaning unwanted touching or grabbing kissing or fondling against the respondent’s will one or more times. PMKs history of exposure to physical IPV was assessed as having seen or heard parents or other caregivers hitting each other or another adult in the home three or more times. PMKs were categorized as having experienced exposure to emotional or verbal IPV if they had experienced having seen or heard parents or other caregivers say hurtful or mean things to one another or another adult in the home six or more times. Exposure to emotional abuse by the PMK was defined as experiencing hurtful things said to them or feeling unwanted or loved six or more times. Physical neglect was defined as not having basic needs, such as being kept clean or having food and clothing provided. PMKs were categorized as having experienced slapping/spanking if a parent or other caregiver spanked the respondent with their hand on the bottom or slapped their hand three or more times before age 16 years.

#### PMK current mental health, substance use and family circumstances

Marital conflict was measured by asking respondents who were in a current relationship how many times in the past 12 months the respondent and their partner have had major disagreements (e.g., “big fights”, “blow-ups”). Respondents were categorized as: 1) no (i.e., *never happened* or *not in the past 12 months*), 2) one or two times, or 3) three times or more. Mental health functioning and emotional well-being was measured using Keyes’ 14-item Mental Health Continuum – Short Form [29–32] to assess flourishing mental health functioning and emotional well-being compared to languishing to moderate mental health functioning and emotional well-being (e.g., feeling happy, interested in life). Flourishing mental health functioning and emotional well-being was defined as high levels (i.e., *almost every day* or *everyday*) on a minimum of one of the three measures of emotional well-being and a minimum of six of 11 measures of positive functioning.

Family functioning was measured using the general functioning subscale of the McMaster Family Assessment Device used in the 1983 OCHS (12 questions with scores ranging from 12 to 48) [33]. Family functioning included items such as asking about supporting family during crisis, talking to each other when sad, accepting each other, discussing fears, solving problems, and communicating with each other. Scores were dichotomized into unhealthy family functioning (scores 26 and above) compared to healthy family functioning (scores below 26). PMKs were asked about current smoking. Individuals who self-reported smoking occasionally to daily were coded as yes to smoking compared to those who did not smoke. For alcohol use, individuals who responded yes to one or more of the following were categorized as having moderate or greater alcohol use: 1) drinking alcoholic beverages four to six times a week or more in the past six months; and 2) having five drinks or more on one occasion four times or more in the past four weeks.

#### Youth reports of slapping/spanking

Youth’s exposure to slapping/spanking was measured by asking youth how often they experienced being spanked with a hand on the bottom (bum) or slapped on the hand by a parent and/or caregiver. Those reporting having been slapped/spanked three or more times were coded as “yes”.

#### Sociodemographic factors

Sociodemographic factors considered in our analyses included PMK sex, family poverty (composite variable derived from family size, population centre, and household income using a dichotomous poverty cut-off), single parenthood (two-parent household, single parent), ethnicity (White, Indigenous, South Asian, East and Southeast Asian, West Asian and Arab, Black, Latin American, and multi-race or other), and area of residence (rural, small to medium urban, large urban).

### Statistical analyses

First, the prevalence of slapping/spanking experiences of the PMK and the youth were computed. Second, descriptive statistics were calculated and unadjusted logistic regressions were computed to examine the relationships of PMK sociodemographic factors and youth reports of slapping/spanking. Third, logistic regression analyses were computed to examine the association between PMK’s childhood experiences and PMK’s current mental health, substance use and family functioning with youth reports of slapping/spanking adjusting for area of residence, PMK’s sex, family poverty, ethnicity, and single parenthood. Statistical sampling weights were applied to ensure representativeness of the population. Bootstrapping was used as a variance estimation method [34].

## Results

The lifetime prevalence of experiencing slapping/spanking was 46.4% among PMK and 18.0% among youth. Compared to living in rural areas, youth in urban areas had decreased odds of experiencing being slapped/spanked. Family poverty was also associated with a decreased odds of youth reported slapping/spanking (see Table 1). PMK’s experiences of physical and verbal bullying victimization, PMK experience of being slapped/spanked in childhood, PMK’s reports of sexual abuse, emotional abuse, and exposure to physical IPV were all associated with increased odds of youth reported slapping/spanking (see Table 2). PMK’s reports of being physically abused and exposed to emotional/verbal IPV as a child were associated with reduced odds of youth reported slapping/spanking. No significant relationships were noted for PMK’s experiences of physical neglect and of youth reported slapping/spanking.

**Table 1:** Prevalence of youth reported slapping/spanking by sociodemographic variables

	Youth Not Slapped/Spanked % (95% CI)	Youth Slapped/Spanked % (95% CI)	OR (95% CI)
Urban			
Rural	75.70 (73.67, 77.59)^a^	24.30 (22.41, 26.33)^a^	1.00
Small and Medium Urban	83.03 (81.02, 84.85)^a^	16.97 (15.15, 18.98)	0.64 (0.53, 0.76)*
Large Urban	82.78 (82.07, 83.63)^a^	17.22 (16.37, 17.93)^a^	0.64 (0.57, 0.73)*
Family Poverty			
No	81.44 (80.67, 82.18)	18.56 (17.82, 19.33)	1.00
Yes	85.95 (84.81, 87.02)	14.05 (12.98, 15.19)	0.72 (0.65, 0.80)*
Single Parent			
No	82.30 (81.55, 83.03)	17.70 (16.97, 18.45)	1.00
Yes	83.22 (81.68, 84.65)	16.78 (15.35, 18.32)	0.94 (0.84, 1.05)

*CI* confidence interval, *OR* odds ratio. Weighted prevalence and mean. ^a^Prevalence based on counts rounded to the nearest 50 due to Research Data Centre vetting rules. * *p* < .001

**Table 2:** Association between parents’ childhood experiences and youth reports of slapping/spanking

	Youth Not Slapped/Spanked % (95% CI)	Youth Slapped/Spanked % (95% CI)	AOR (95% CI)
Physical Bullying	75.32 (72.51, 77.94)	24.68 (22.06, 27.49)	1.61 (1.38, 1.88)**
Verbally Bullying	78.51 (76.93, 80.00)	21.49 (20.00, 23.07)	1.38 (1.24, 1.53)**
Slapping/Spanking	78.10 (77.04, 79.13)	21.90 (20.87, 22.96)	1.63 (1.48, 1.80)**
Physical Abuse	85.87 (84.48, 87.16)	14.13 (12.84, 15.52)	0.72 (0.64, 0.82)**
Sexual Abuse	77.21 (75.40, 78.93)	22.79 (21.07, 24.60)	1.33 (1.19, 1.49)**
Emotional Abuse	75.57 (73.57, 77.47)	24.43 (22.53, 26.43)	1.75 (1.55, 1.99)**
Exposure to Physical IPV	74.92 (72.22, 77.43)	25.08 (22.57, 27.78)	1.77 (1.52, 2.07)**
Exposure to Emotional/Verbal IPV	83.54 (82.13, 84.84)	16.46 (15.15, 17.87)	0.88* (0.79, 0.9995)
Physical Neglect	82.18 (80.00, 84.18)	17.82 (15.82, 20.00)	0.99 (0.84, 1.17)

*CI* confidence interval, *AOR* adjusted odds ratio, *IPV* intimate partner violence. AOR adjusts for person most knowledgeable (PMKs)’ sex, urbanicity, family poverty, ethnicity, and single parenthood. *p < .05, **p < .001

The findings for the relationships between PMK’s current mental health, substance use and family circumstances and youth reported slapping/spanking are presented in Table 3. Experiencing low levels of marital conflict compared to no marital conflict was associated with decreased odds of youth reported slapping/spanking, while higher levels of marital conflict compared to those with no marital conflict were associated with an increased likelihood of youth reported slapping/spanking. PMK’s reports of languishing to moderate mental health (compared to flourishing) and moderate or greater alcohol use were associated with increased odds of youth reported slapping/spanking. Significant relationships were not found for family functioning or smoking and youth reported slapping/spanking.

**Table 3:** Associations between parents’ current mental health, substance use and family circumstances and youth self-reports of slapping/spanking

	Youth Not Slapped/Spanked % (95% CI)	Youth Slapped/Spanked % (95% CI)	AOR (95% CI)
Marital Conflict			
0 times	82.79 (81.86, 83.68)	17.21 (16.32, 18.14)	1.00
1 or 2 times	84.80 (83.57, 85.95)	15.20 (14.05, 16.43)	0.80 (0.72, 0.89)*
3+ times	77.32 (74.58, 79.84)	22.68 (20.16, 25.42)	1.43 (1.21, 1.69)*
Mental Health Functioning and Emotional Well-Being^a^			
Flourishing	83.27 (82.49, 84.01)	16.73 (15.99, 17.51)	1.00
Moderate to Languishing	75.60 (73.80, 77.31)	24.40 (22.69, 26.20)	1.61 (1.43, 1.82)*
Smoking			
No	82.62 (81.90, 83.31)	17.38 (16.69, 18.10)	1.00
Yes	83.33 (81.49, 85.02)	16.67 (14.98, 18.51)	0.98 (0.86, 1.13)
Moderate or Greater Alcohol Use			
No	82.61 (81.88, 83.31)	17.39 (16.69, 18.12)	1.00
Yes	78.16 (75.79, 80.35)	21.84 (19.65, 24.21)	1.36 (1.16, 1.58)*
Family Functioning^b^			
Healthy	82.42 (81.67, 83.13)	17.58 (16.87, 18.33)	1.00
Unhealthy	81.56 (79.83, 83.18)	18.44 (16.82, 20.17)	1.03 (0.90, 1.18)

*CI* confidence interval, *AOR* adjusted odds ratio, AOR adjusts for person most knowledgeable (PMKs)’ sex, urbanicity, family poverty, ethnicity, and single parenthood. ^a^Mental health functioning and emotional well-being measured using Keyes’ 14-item Mental Health Continuum – Short Form; ^b^Family functioning was measured using the general functioning subscale of the McMaster Family Assessment Device. *p < .001

## Discussion

This study identified several unique findings regarding experiences of slapping and spanking. First, several childhood experiences of the PMK including physical and verbal bullying by peers, sexual abuse, emotional abuse and exposure to physical IPV were associated with increased odds of youth reports of slapping/spanking. Second, higher frequency of marital conflict was associated with an increased likelihood of youth reported slapping/spanking, while low levels of marital conflict were protective against slapping/spanking. Third, a PMK having languishing or moderate mental health functioning and emotional well-being compared to flourishing was associated with increased odds of youth reports of slapping/spanking. Finally, PMK alcohol use was associated with an increased likelihood of youth reports of slapping/spanking.

Interestingly, the prevalence of slapping/spanking was higher for PMK (46%) compared to youth (18%). Although it is encouraging that the prevalence of slapping/spanking was reported as lower for youth, it is not possible to determine from these data if slapping/ spanking has decreased across generations. Consistent with previous research, a PMK’s history of being slapped/spanked was associated with an increased likelihood of youth reports of slapping/spanking [35] and a PMK’s report of experiencing physical abuse was associated with decreased odds of youth reported slapping/spanking [20]. Importantly, the current study extends knowledge beyond attitudes to examine slapping/spanking behaviours. This finding may seem counterintuitive as one might expect that those growing up experiencing violence within the home may be more tolerant of violence as they become parents. It may also reflect individuals not wanting to pass on the experience of physical abuse to their own children and therefore making the decision to avoid use of any physical force including spanking [36]. Further research is necessary to confirm this finding and to determine the underlying mechanisms, which could inform prevention strategies.

Studies of child protection investigations have shown that many cases of physical abuse result from a parent’s attempt to discipline children using corporal punishment going too far [37]. This implies that preventing spanking could be one means of reducing the occurrence of physical abuse. Our finding that a PMK’s emotional abuse experience was associated with an increased likelihood of youth reports of slapping/spanking was not consistent with previous work on spanking attitudes [20]. This may indicate that emotional abuse experiences of the parents are an important consideration for prevention efforts.

Research on child maltreatment at home and bullying by peers is emerging [38]. What is not well known is how a parent’s past experiences of bullying victimization may be linked to later parenting such as spanking. Since PMK’s childhood experiences of verbal and physical bullying were associated with increased odds of youth’s reports of slapping/spanking, current findings may indicate that the prevention of bullying victimization could have benefits for families with the possible decrease of slapping/spanking exposure for the next generation. Prevention efforts to reduce bullying may be an example of an upstream public health approach that aims to change the culture of tolerance for violence against children within North America, which could extend into the family and be consistent with efforts of the UN Convention on the Rights of the Child.

In the current study poverty was found to be associated with decreased odds of slapping/spanking. More research is needed to replicate and clarify this relationship with regards to the underlying mechanisms. It may be that families living in poverty in the current study are receiving additional supports and resources that moderate the relationship between poverty and slapping/spanking. Ontario Early Years Centres and Public Health Units that offer parenting support programs, such as Triple P, that would address positive parenting strategies. It was not possible to examine this hypothesis with the current data. In the current study, family functioning generally assessed support, problem solving, communication, and compatibility between family members. Interestingly, unhealthy family functioning was not associated with slapping/spanking. Current findings also showed that high marital conflict (major arguments three or more times over the last 12 months), poorer PMK mental health and well-being, and moderate or greater PMK alcohol use were all related to youth reports of slapping/spanking. It has been found that parenting stress is associated with an increased likelihood of harsh parenting including spanking [15, 39]. Maternal marital satisfaction has been found to attenuate the relationship between parenting stress and spanking [39]. This provides further support for the need to examine how family relationships and mental health functioning and emotional well-being of parents can be improved as a way to create a healthier home environments for children. In addition, it may be important for prevention strategies to include strategies for reducing parental alcohol use since greater use of alcohol has been associated with increased likelihood of physical and emotional abuse [40] and was associated with slapping/spanking in the current study. Future work is needed to further understand if reducing marital conflict and parental alcohol use and improving mental health functioning and emotional well-being among parents, aside from the obvious benefits for family members, may be an effective way to prevent or reduce the use of slapping/spanking.

This study has several limitations. First, it is cross-sectional so inferences about causation cannot be determined. Second, data on slapping/spanking are retrospective. However, evidence does indicate that retrospective recall of adverse childhood experiences provides valid and reliable data [41–43]. Third, only one item was used to assess slapping/spanking in the current study. The means that we were unable to separate the experiences of being spanked with a hand on the bum and being slapped on one’s hand. Fourth, the relationship of the person who slapped/spanked the adolescent was unknown, which means it may or may not have been the PMK. Regardless, the PMK was either the person committing the slapping/spanking or was in a caregiver role in a family environment or circumstances where slapping/spanking was occurring. Finally, smoking was assessed with only one item that did not provide great detail with regards to the specific consumption based on the response categories of daily, occasionally, and not at all. Further work using more detailed measures of smoking is required before conclusions regarding smoking and slapping/spanking can be made.

## Conclusions

With recommendations that children should not be spanked [10, 11], understanding what is related to slapping/spanking is needed to inform prevention efforts. Our current findings identify key factors that are related to an increased likelihood of youth reported slapping/spanking. Programs and strategies to prevent spanking are emerging with varying degrees of evidence for proven effectiveness [44]. Efforts to prevent slapping/spanking could be expanded to take into account how a broad range of parental experiences (i.e., past bullying experiences, child maltreatment history, marital conflict, moderate or languishing mental health functioning and emotional well-being, and alcohol use) that influence the use of slapping/spanking. From a global perspective, preventing violence against children is a difficult task, but one that is necessary to conform to the UN Convention on the Rights of children and for the success of the UN Sustainable Development Goals. At the individual-level, the prevention of slapping/spanking is needed to give each child the opportunity for healthy development.

## Data Availability

Data can be accessed with permission from Statistics Canada. The data can be accessed through the Statistics Canada Research Data Centre. A formal process is in place for researchers to apply for access to the data only within a Statistics Canada Research Data Centre.

## Abbreviations

IPV: Intimate partner violence
OCHS: 2014 Ontario Child Heath Study
PMK: Person most knowledgeable

## References

[1] Donnelly M, Straus MA (2005). Corporal punishment of children in theoretical perspective.

[2] Gershoff ET (2002). Corporal punishment by parents and associated child behaviors and experiences: a meta-analytic and theoretical review. Psychol Bull.

[3] Gershoff ET, Grogan-Kaylor A (2016). Spanking and child outcomes: old controversies and new meta-analyses. J Fam Psychol.

[4] Durrant JE, Ensom R (2012). Physical punishment of children: lessons from 20 years of research. CMAJ..

[5] Afifi TO, Brownridge DA, Cox BJ, Sareen J (2006). Physical punishment, childhood abuse and psychiatric disorders. Child Abus Negl..

[6] Afifi TO, Mota NP, Dasiewicz P, MacMillan HL, Sareen J (2012). Physical punishment and mental disorders: results from a nationally representative US sample. Pediatrics..

[7] Afifi TO, Ford D, Gershoff ET (2017). Spanking and adult mental health impairment: the case for the designation of spanking as an adverse childhood experience. Child Abus Negl.

[8] Merrick MT, Ports KA, Ford DC, Afifi TO, Gershoff ET, Grogan-Kaylor A (2017). Unpacking the impact of adverse childhood experiences on adult mental health. Child Abuse Negl.

[9] MacMillan HL, Boyle MH, Wong YY, Duku EK, Fleming JE, Walsh CA (1999). Slapping and spanking in childhood and its association with lifetime prevalence of psychiatric disorders in a general population sample. CMAJ..

[10] Sege RD, Siegel BS, Council on Child Abuse and Neglect, Committee on Psychosocial Aspects of Child and Family Health. Effective Discipline to Raise Healthy Children. Pediatrics. 2018;e20183112. http://pediatrics.aappublications.org/content/early/2018/11/01/peds.2018-3112.abstract. 10.1542/peds.2018-3112 30397164

[11] Afifi TO, Romano E (2017). Ending the spanking debate. Child Abuse Negl.

[12] Giles-Sims J, Straus MA, Sugarman DB (1995). Child, maternal, and family characteristics associated with spanking. Fam Relat.

[13] Wissow LS (2001). Ethnicity, income, and parenting contexts of physical punishment in a national sample of families with young children. Child Maltreat.

[14] Huang C-C, Lee I (2008). The first-three years of parenting: evidence from the fragile families and child well-being study. Child Youth Serv Rev.

[15] MacKenzie MJ, Nicklas E, Brooks-Gunn J, Waldfogel J (2011). Who spanks infants and toddlers? Evidence from the fragile families and child well-being study. Child Youth Serv Rev.

[16] Perron JL, Lee CM, Laroche KJ, Ateah C, Clément M-È, Chan K (2014). Child and parent characteristics associated with Canadian parents’ reports of spanking. Can J Community Ment Heal.

[17] Grogan-Kaylor A, Burlaka V, Ma J, Lee S, Castillo B, Churakova I (2018). Predictors of parental use of corporal punishment in Ukraine. Child Youth Serv Rev.

[18] Grogan-Kaylor A, Otis MD (2007). The predictors of parental use of corporal punishment. Fam Relat.

[19] Combs-Orme T, Cain DS (2008). Predictors of mothers’ use of spanking with their infants. Child Abuse Negl.

[20] Gagné M-H, Tourigny M, Joly J, Pouliot-Lapointe J (2007). Predictors of adult attitudes toward corporal punishment of children. J Interpers Violence.

[21] Bell T, Romano E (2012). Opinions about child corporal punishment and influencing factors. J Interpers Violence..

[22] Wilson LM, Lorber MF, Timmons Fritz PA (2018). A dynamic approach to risk factors for maternal corporal punishment in early to middle childhood. J Child Fam Stud.

[23] Regalado M, Sareen H, Inkelas M, Wissow LS, Halfon N. Parents’ discipline of young children: Results from the National Survey of Early Childhood Health. Pediatrics. 2004;113(Supplement 5):1952–1958. http://www.ncbi.nlm.nih.gov/pubmed/9521967. Accessed 8 June 2018. 15173466

[24] Davis RN, Davis MM, Freed GL, Clark SJ (2011). Fathers’ depression related to positive and negative parenting behaviors with 1-year-old children. Pediatrics..

[25] Lee SJ, Perron BE, Taylor CA, Guterman NB (2011). Paternal psychosocial characteristics and corporal punishment of their 3-year-old children. Artic J Interpers Violence.

[26] MacMillan HL, Boyle MH, Taillieu T, Cheung K, Sareen J, Afifi TO (2014). Child abuse and mental disorders in Canada. Can Med Assoc J.

[27] Walsh CA, MacMillan HL, Trocmé N, Jamieson E, Boyle MH (2008). Measurement of victimization in adolescence: development and validation of the childhood experiences of violence questionnaire. Child Abuse Negl.

[28] Eunice Kennedy Shriver National Institute of Child Health and Human Development, University of North Carolina Carolina Population Centre. Add Health Codebook Explorer (ACE): Topic: Mistreatment by adults. The National Longitudinal Study of Adolescent to Adult Health. https://www.cpc.unc.edu/projects/addhealth/documentation/ace/tool/topic?TopicId=172. Accessed 26 Nov 2018.

[29] Keyes CLM (2006). Mental health in adolescence: is America’s youth flourishing?. Am J Orthop.

[30] Keyes CLM (2007). Promoting and protecting mental health as flourishing: a complementary strategy for improving national mental health. Am Psychol.

[31] Keyes CLM (2005). Mental illness and/or mental health? Investigating axioms of the complete state model of health. J Consult Clin Psychol.

[32] Keyes CLM. Atlanta: Brief description of the mental health continuum short form (MHC-SF). https://www.aacu.org/sites/default/files/MHC-SFEnglish.pdf.

[33] Byles J, Byrne C, Boyle MH, Offord DR (1988). Ontario child health study: reliability and validity of the general functioning subscale of the McMaster family assessment device. Fam Process.

[34] Statistics Canada. Microdata USER GUIDE: 2014 Ontario Child Health Study 2017.

[35] Barkin S, Scheindlin B, Ip EH, Richardson I, Finch S (2007). Determinants of parental discipline practices: a national sample from primary care practices. Clin Pediatr (Phila).

[36] Ertem IO, Leventhal JM, Dobbs S (2000). Intergenerational continuity of child physical abuse: how good is the evidence?. Lancet..

[37] Durrant JE, Trocmé N, Fallon B, Milne C, Black T, Knoke D. Punitive Violence against Children in Canada. Toronto, On; 2006. www.cecw-cepb.ca/infosheets. .

[38] Vikse Nicholson J, Chen Y, Huang C-C (2018). Children’s exposure to intimate partner violence and peer bullying victimization. Child Youth Serv Rev.

[39] Liu L, Wang M (2015). Parenting stress and harsh discipline in China: the moderating roles of marital satisfaction and parent gender. Child Abuse Negl.

[40] Kepple NJ (2018). Does parental substance use always engender risk for children? Comparing incidence rate ratios of abusive and neglectful behaviors across substance use behavior patterns. Child Abuse Negl.

[41] Hardt J, Vellaisamy P, Schoon I (2010). Sequelae of prospective versus retrospective reports of adverse childhood experiences. Psychol Rep.

[42] Hardt J, Rutter M (2004). Validity of adult retrospective reports of adverse childhood experiences: review of the evidence. J Child Psychol Psychiatry.

[43] Hardt J, Sidor A, Bracko M, Egle UT (2006). Reliability of retrospective assessments of childhood experiences in Germany. J Nerv Ment Dis.

[44] Gershoff ET, Lee SJ, Durrant JE (2017). Promising intervention strategies to reduce parents’ use of physical punishment. Child Abuse Negl.

